# Supplementary material for: ProteinShader: illustrative rendering of macromolecules
Source: BMC Struct Biol. 2009 Mar 30;9:19. doi: 10.1186/1472-6807-9-19 (PMC2672931; doi:10.1186/1472-6807-9-19)
Supplement: Additional file 1 — ProteinShader program without source code. This compressed file contains the complete ProteinShader program including associated libraries, but no source code. A README.txt file gives an overview of the ProteinShader distribution, and the index.html file in the help subdirectory has directions on getting started with the program as well as a set of tutorials. [file 1472-6807-9-19-S1.zip › ProteinShader-beta-0_9_4-binary/help/tutorials/1HGU.html]

Tutorial: 1HGU and 3HHR


# ProteinShader Tutorial


♦ Home


♦ Download


♦ Getting Started

Choose a Link:

Installation

Running

Troubleshooting

PDB Files


♦ Tutorials

Choose a Link:

List of Proteins

Style Options

Saving Images


♦ General Controls

Choose a Link:

Menu Bar

Keyboard Shortcuts

Mouse Motion

Motion Panel

Antialiasing


♦ Cartoon Controls

Choose a Link:

Control Panels

Selection Panel

Radio Buttons

Decorations Panel

Adding Textures

Color Panel

Visibility Panel

Side Chains Panel


♦ Atom Controls

Choose a Link:

Control Panels

Selection Panel

Radio Buttons

Color Panel

Visibility Panel

Scale Panel

Tiling Panel


♦ Developers

Choose a Link:

Compiling

Running

Code Docs

Developer Links


## Human Growth Hormone: Four Alpha-Helix Bundle [PDB:1HGU]

The core of the hGH (human growth hormone) protein is a four-helix bundle
structure comprised of two pairs of parallel alpha-helices that are joined in an
antiparallel fashion [1]. X-ray crystallography has
been used to determine the three-dimensional structure of the hGH protein by
itself
[2, 3]
and complexed with the extracellular domain of its receptor
[4, 5].
To load the hGH protein structure, select Open from the
File menu
of the ProteinShader program and then use the file chooser box to select the
1HGU.pdb file.


## Cartoon-style displays with side chains

When the hGH protein is first loaded, it will be displayed as a pen-and-ink
style drawing of ribbons and tubes. To switch to an all tubes style display as
shown in Figure 1A, go to the
Style menu
above the canvas and select Tubes from the Cartoon submenu. The tubes
representing alpha-helices have a thicker diameter and are drawn a little more
darkly than the loop regions connecting them. The amino-terminus of the
polypeptide chain is seen at the top left of the figure, while the
carboxyl-terminus is near the top center. What appears to be a chain
termination near the bottom right of Figure 1A is in fact a gap in the
structure. Amino acid residues 37 to 39 are disordered in the 1HGU crystal
structure, presumably because they are part of a flexible loop that is moving
around in the solvent that makes up part of the crystal. When data on atom
positions is missing from a PDB file, the ProteinShader program does not
attempt to fill in missing parts of a tube or ribbon, but rather simply puts
end caps on the tube or ribbon before and after the gap.

Amino acid side chains that strongly modulate binding of hGH to its receptor
have been identified by a strategy known as alanine-scanning mutagenesis
[6]. To illustrate these side chains as shown in
Figure 1B, use the menu at the top right of the Control Panel to switch from the
Decorations subpanel
to the
Cartoon Side Chains subpanel.
Then go to the list of amino acids on the left side of the Control Panel
and left-click with the mouse to select residues number 10, 54, 56, 58, 64, 68,
171, 172, 174, 175, 176, 178, 182, and 185 while holding down the keyboard Ctrl
key (command key on Macintosh). When the first of these residues was clicked
on, the menu under the Selected radio button near the top of the Control Panel
should have automatically switched to Residues, so clicking the Space Filling
button should now cause the side chains of the selected residues to appear as
spheres. By default, the spheres are colored by atom type. To color by amino
acid type, switch from the
Cartoon Side Chainssubpanel
to the
Atom Color subpanel
and use the Amino Acid button at the very bottom of the subpanel.

To get a better look at the spatial arrangement of the sides chains that affect
receptor binding, use the left-side mouse button to drag the mouse across the
canvas and rotate the image to a position similar to Figure 1C (the protein can
be returned to its original orientation at any time by using the
Orientation menu).
For a more schematic view of the side chains, go back to the
Cartoon Side Chains subpanel,
and, after making sure that Residues is still selected in the
menu under the Selected radio button, click on the Balls and Sticks button.
To color the balls by atom type, go back to the
Atom Color subpanel
and use the Atom Type button. To obtain an image similar to Figure 1D,
use the
Style menu
above the canvas to select Ribbons from the Cartoon submenu, and then go back
to the
Decorations subpanel,
choose Model from the menu under the Selected radio button, and select
Hatching 1 from the Bend Texture menu.

In addition to rotating the image with the mouse, the center mouse button or
scroll-wheel can be used to zoom in and out on the image, while the right-side
mouse button can be used for moving the image up and down or side to side
(see
Mouse Motion Control
section for details and for Macintosh single-button mouse options). By using
these mouse manipulations, it should be fairly easy to obtain a closeup view
of the side chains similar to Figure 2.


|  |  |
| --- | --- |
| **A** | **B** |
| **C** | **D** |

**Figure 1.** Illustration of hGH side chains that affect receptor binding.
(A) hGH rendered as tubes with halftoning. (B) The same image as part A, except
that amino acid side chains implicated in receptor binding are shown as a space
filling (spheres) style display with colors based on amino acid type. (C) The
same image as part B, except that the protein has been rotated to get a better
look at the side chains. (D) The protein is shown in the same orientation as in
part C, but using ribbons rather than tubes and with side chains shown as a
balls and sticks style display with colors based on atom type.
The images were captured as described in the
Saving Images
section of the tutorials main page.


**Figure 2.** Closeup view of amino acid side chains that modulate hGH
receptor binding. The image is the same as in Figure 1D, except that mouse
controls have been used to zoom in on a region of the protein.
The images were captured as described in the
Saving Images
section of the tutorials main page.


## hGH bound to receptor extracellular domains

Binding of hGH to its cognate receptor is required for regulation of normal
human growth and development, and this binding appears to activate signal
transduction through a mechanism that involves dimerization of receptor
molecules [4, 7].
The structure of hGH complexed with two molecules of the extracellular domain
of its receptor has been solved by x-ray crystallography
[4, 5], and can be loaded
into the ProteinShader program by using the
File menu
above the canvas to open the 3HHR.pdf file.

When the structure is first opened, it will be shown as a ribbons and tubes
style display. To obtain an orientation similar to Figure 3A, select Right
from the
Orientation menu
above the canvas, and then drag the mouse vertically downwards a short distance.
Beta-strands, which dominate the receptor extracellular domain, are shown as
wide ribbons, while alpha-helices, which dominate the hGH structure, are shown
as tubes. To place stripes on the beta-strands, go to the
Decorations subpanel
and click on the β-Strands radio button to select all beta-strands, and
then select Vertical Bars from the Halftoning Texture menu and None from the
Bend Texture menu.

To fade out the loop regions (thin ribbons) between
alpha-helices and beta-strands, go to the menu at the top right of the Control
Panel and change it from the
Decorations subpanel
to the
Cartoon Visibility subpanel. Click on the Loops radio button to select
all loops, and then click on the Translucent button. The default value for
translucency is 75%, but can be adjusted by using the slider control below
the Translucent button. To explore the three-dimensional structure, use the
Mouse Motion Controls
and the
Orientation menu
above the canvas.

The hGH amino acid side chains illustrated in Figures 1 and 2 define the high
affinity receptor binding site, Site1, which binds the receptor first, after
which a second lower affinity site on the other side of hGH, Site2, can bind
a second copy of the receptor
[8].
To see where the Site1 side chains
contact the receptor, try using the Cartoon Side Chains subpanel as described
above for Figure 1B. It may also be helpful to experiment with visibility
status, decorations, and color changes by using the
Cartoon Visibility,
Decorations, and
Cartoon Color
subpanels.


|  |  |
| --- | --- |
| **A** | **B** |

**Figure 3.** The hGH protein is shown bound to the extracellular domain
of its receptor, hGHbp. (A) A ribbons and tubes style display of the hGH
protein bound to two molecules of hGHbp. The two beta-strand dominated domains
on the left half of the image are one copy of hGHbp, while the two beta-strand
dominated domains on the right half are the second copy. The view shows one
end of the four-helix bundle that forms the core of the hGH protein.
(B) Same image as in part A, but after rotating it approximately
90 degrees to get a side view of the four-helix bundle.
The images were captured as described in the
Saving Images
section of the tutorials main page.


## References

1.
Brandon C, Tooze J: **Alpha-Domain Structures.**
In *Introduction to Protein Structure.* 2nd edition.
New York: Garland Publishing; 1998: 35-46.

2.
Chantalat L, Jones ND, Korber, F, Navaza, J, Pavlovsky AG:
**The crystal-structure of wild-type growth-hormone
at 2.5 angstrom resolution.**
*Protein Pept Lett* 1995, **2**: 333-340.

3.
**Human growth hormone PDB entry 1HGU**
[http://www.rcsb.org/pdb/explore/explore.do?structureId=1HGU]

4.
de Vos AM, Ultsch M, Kossiakoff AA:
**Human growth hormone and extracellular domain of its receptor:
crystal structure of the complex.**
*Science* 1992, **255**: 306-312.

5.
**Human growth hormone and extracellular domain of its receptor
PDB entry 3HHR**
[http://www.rcsb.org/pdb/explore.do?structureId=3HHR]

6.
Cunningham BC, Wells JA:
**High-resolution epitope mapping of hGH-receptor interactions by
alanine-scanning mutagenesis.**
*Science* 1989, **244**: 1081-1085.

7.
Brandon C, Tooze J: **Signal Transduction.**
In *Introduction to Protein Structure.* 2nd edition.
New York: Garland Publishing; 1998: 251-281.

8.
Kossiakoff AA:
**The structural basis for biological signaling, regulation, and specificity
in the growth hormone-prolactin system of hormones and receptors.**
*Adv Protein Chem* 2004, **68**:147-169.


---

|  |  |  |  |
| --- | --- | --- | --- |
| Hosted by |  |  |  |

Webmaster: Joe Weber,
joe.weber AT alumni.duke.edu
  
Web site last updated: December 30th, 2008
  
© 2007–2008 Joseph R. Weber
